# Posttraumatic growth and recovery among a sample of Egyptian mental health service users: a phenomenological study

**DOI:** 10.1186/s12888-022-03919-x

**Published:** 2022-04-12

**Authors:** Nashwa Ibrahim, Fiona Ng, Abeer Selim, Eman Ghallab, Amira Ali, Mike Slade

**Affiliations:** 1 Psychiatric and Mental Health Nursing Department, Faculty of Nursing, Mansoura University, Mansoura, Egypt; 2 School of Health Sciences, Institute of Mental Health, University of Nottingham, Nottingham, UK; 3 College of Nursing, King Saud Bin Abdulaziz University for Health Sciences, Riyadh, Saudi Arabia; 4 King Abdullah International Medical Research Center, Riyadh, Saudi Arabia; 5 Nursing Education Department, Faculty of Nursing, Alexandria University, Alexandria, Egypt; 6 Tokyo University of Agriculture and Technology, Fuchu, Japan; 7 Department of Psychiatric Nursing and Mental Health, Faculty of Nursing, Alexandria University, Alexandria, 21527 Egypt; 8 Nord University, Postboks 474, 7801 Namsos, Norway

**Keywords:** Mental health recovery, Service-user accounts, Low-middle-income countries, Posttraumatic growth, Qualitative research

## Abstract

**Background:**

Delivery of recovery-oriented mental health practice is fundamental to personal recovery. Yet, there is lack of service users’ accounts on what constitutes mental health recovery in Egypt.

**Objectives:**

The aim of this study was to explore mental health recovery meaning informed by people with personal experience of recovery.

**Methods:**

A phenomenological research design was used. Semi-structured qualitative interviews were conducted with 17 adult community-dwelling individuals who identified as recovered/recovering from mental health issues. An inductive thematic analysis approach was used to analyses participants’ responses.

**Results:**

Participants predominately reported personal and functional definitions of mental health recovery. Posttraumatic growth was the strongest theme comprising: relation to others, spirituality, new possibilities, identity & strengths, and appreciation of life. Themes of acceptance and forgiveness, functional and clinical recovery, and finding hope were also identified.

**Conclusions:**

This is the first study to explore mental health recovery meaning among a sample of people with lived experience of mental health issues in Egypt. Findings suggest that developing and implementing psychosocial interventions to support posttraumatic growth among people with mental health issues is a priority.

## Introduction

Improving mental health services in Low and Middle Income Countries (LAMICs) is both an economic and moral imperative [1, 2]. A nationwide Egyptian community survey revealed that the prevalence of mental health issues is approximately 17% of the adult population [3]. Egyptian mental health services predominately use a biomedical approach to mental health practice [3, 4], where service users report restrictions to their basic human rights impeding both the quality of care received and their recovery [5]. Despite significant increases in Egyptian people seeking help, community mental health services across Egypt remain significantly under-resourced [6]. 

Ensuring service user representation is critical to improving mental health services [7]. Initiatives such as person-centered care, the recovery model, and user involvement have contributed to substantial changes in mental health systems in Anglophone countries [8]. One of the challenges to achieving social justice in health care in Egypt is the lack of representation of service users in health care delivery [9].

Studies report positive psychological changes following the experience of traumatic events as mental health issues and are often part of mental health recovery. Calhoun, Cann, Tedeschi, and McMillan defined PTG as “the experience of significant positive change arising from the struggle with a major life crisis” [10]. These changes are usually referred to as posttraumatic growth (PTG) [11, 12]. Individuals who survive trauma or highly stressful experiences undergo high levels of emotional distress which could be managed by individuals’ adaptive coping mechanisms. The cognitive engagement and processing of traumatic or stressful experiences are associated with PTG [13].

Differences between clinicians and service users’ definitions of mental health recovery reflects the complexity and multidimensional nature of recovery [14]. A qualitative study exploring the perspectives of Egyptian mental health professionals on mental health recovery reported that functional recovery outweighed other definitions [15]. Functional recovery refers to carrying out daily living activities, social relationships, and vocational and educational activities [16]. Yet, people with lived experience place emphasis on personally meaningful experiences which can occur with or without symptom amelioration as described in the study conducted in South Africa [17] and the review of the international definitions of mental health recovery [18]. Recovery, on the other hand, has been defined as a “deeply personal, unique process of change… a way of living a satisfying, hopeful and contributing life even with limitations caused by illness [and] a process involving the development of new meaning or purpose in one’s life” [19].

Insufficient data exists regarding the perspectives of Egyptian mental health service users on recovery [20]. There is a need to operationalize the concept of personal recovery to translate policies into practices and deliver consumer-centered care in Egypt [21]. The current study aims to explore the meaning of mental health recovery informed by people with mental health issues living in the community in Egypt.

## Methods

### Design

A qualitative phenomenological research design [22] was used where participants were asked to describe their lived experience [23] about mental health recovery. Phenomenology enables researchers to develop deep understanding of a phenomena under investigation [24].

### Participants

Adults living in the community with mental health issues who self-identify as having recovery experiences were eligible to participate in the study (diagnoses were made by their treating psychiatry specialists/consultants). Individuals with neurodevelopmental disorders, substance use disorders, and organic mental disorders were excluded. Individuals did not have to be engaged in mental health services to be included in the study.

### Procedures

Faculty of Nursing, Mansoura University Research Ethics Committee approval was obtained prior to commencing with the study (Ref. No. P.0215). A semi-structured topic guide was developed and included questions about definitions e.g. “how would you define mental health recovery?”, meaning, and indicators of mental health recovery e.g. “how would you know if you experienced mental health recovery?”. Prompts were used when necessary to elicit participants’ responses e.g. “would you please give an example to that?”. Two pilot interviews were conducted to ensure clarity of the questions. All interviews were conducted by the first author in informal Egyptian Arabic. Participants were recruited via social media platforms (Facebook and Twitter) and through advertising in out-patient mental health facilities. Participants were interviewed face-to-face or via video conferencing (e.g. Zoom) according to their preference and convenience. All participants received a study information sheet (via email for online interviews) and provided written consent (or verbal consent for online interviews). All participants consented for their data to be used for research purposes. The average length of interviews was 45–50 min. Field notes were taken during interviews to support researcher’s reflection and to support the data analysis [25]. Recruitment and interviewing took place until no new patterns emerged and data saturation was reached [26, 27]. Interviews were recorded, transcribed and translated to English (for analysis and publication purposes) by the first author. To check the quality of translation, four transcripts were back translated to Arabic by one independent co-author (AA), then all translations were reviewed by a professional English Translator.

### Analysis

An inductive six-stage thematic analysis approach (the analysis was driven by the content of the data) was followed [28]. First, the lead author familiarized herself with the data by reading all the transcripts of the interviews and listening to interviews’ recordings twice. Second, initial line-by-line coding of all transcripts was conducted in NVivo 12. Third, codes that shared a unifying feature were collapsed and clustered into themes. Fourth, an independent researcher (EG) checked the developed themes against codes and the entire data set to check whether there was sufficient data to support themes, and to identify the quality of each theme. This process resulted in the amalgamation of some themes into one coherent theme. Fifth, themes were provided labels and definitions. Sixth, themes were interpreted in light of the research question.

### Researcher’s position and reflexivity

Before embarking on the current study, the first author had some predispositions and assumptions about how mental health service users would define recovery. Particularly with first author’s knowledge of mental health services in Egypt which is based on the medical model. The author assumed that service users would emphasize clinical aspects of recovery over personal recovery. During the interviews, the researcher maintained field notes which provided a reflective space for her earlier pre-assumptions; particularly when clinical recovery was the least mentioned themes. This was discussed with one psychiatrist colleague who informed the researcher that he sees many recovery stories that emphasize personal meanings of recovery during his practice, however these stories need to be documented and heard. During transcription and data analysis and specially when domains of PTG emerged, the researcher felt a strong need to explore service users’ stories about mental health recovery in future research.

## Results

### Participants’ characteristics

In total, 17 individuals participated in the study representing three governorates in Egypt (residence included both urban and rural backgrounds). Participants’ characteristics are reported in Table 1.

**Table 1:** Participant characteristics (*N* = 17)

Characteristic	N (%)
Age (years, mean, Standard Deviation)	32.5 ± 9.2 years
Gender	
Female	8 (47.05)
Male	9 (52.94)
Education	
University education	10 (58.82)
Secondary level education	6 (35.29)
Participants with no formal education	1 (5.88)
Diagnoses	
Schizophrenia	4 (23.52)
Borderline personality disorder	3 (17.64)
Depression	3 (17.64)
Bipolar disorder	3 (17.64)
Obsessive compulsive disorder	2 (11.76)
Posttraumatic stress disorder	1 (5.88)
Schizoaffective disorder	1 (5.88)

### Service user accounts on mental health recovery

Five themes emerged: domains of posttraumatic growth, acceptance and forgiveness, functional recovery, hope, and clinical recovery. The coding framework is presented in Table 2.

**Table 2:** Coding framework of mental health recovery

Main theme	Examples
**A. Domains of Post-traumatic growth** A.1 Relating to others	**“**My relationship with my sister and my niece makes me happy, I have no children, my niece calls me mama” (#15) “There was a psychiatrist who taught me in the church, he helped me, he knows about my illness, he supported me, checked on me regularly, and he motivated me. All that was for free” (#8) “My sister Nora is very close to me; I trust her very much” (≠ 1) “I now have compassion for others, I can connect with people, I was arrogant before the experience of mental illness. I now help people and engage in volunteer activities, the pain I went through gave me empathy for others” (≠ 8) “I learned how to be assertive, how to set boundaries and how to express my feelings without shame” (#3) “Recovery means being assertive and being able to defend the self from abuse or harm” (#5)
A.2 Spirituality	“Being a believer has a huge impact on me, it calmed me down after the constant anxiety and fear which were massive after discharge from the hospital” (#1) “I was scared to deal with people, I had no idea what to do in life and work. After my spiritual relation with God, these fears haven’t disappeared completely but were less” (#13) “I turned out to believe that I shouldn’t worry too much about everything, all what I should do is to work hard, God is there for me” (#6) “The spiritual and religious practice have helped me in my journey so much” (#4) “I’m close to god, I do the prayers that have helped me in my journey with the mental health problem” (#8) “ religion gave me serenity” (#16.)
**A.**3 New possibilities	“My experience with the mental health issue made me see my options in life from a different prospective; I can now see the opportunities I have and I can use it all” (#8) “The experience of mental illness made me mature, if you look at my life in 2017, I was staying at home, although I had achievements like admitting to medical license exam step (1), but now is the best stage, I am realizing my dream, my social life is very satisfying” (#9) “I would not achieve what I have now without passing through what happened to me, without the trauma, my life would remain on the old pattern I had before the experience of mental illness” (#1) “The experience of mental illness made me mature, if you look at my life in 2017, I was staying in home, although I had achievements like admitting into the medical license step 1 exam, but now is the best stage, I am realizing my dream, my social life is very satisfying” (#13) “so I would not achieve what I have now without passing through what happened to me, without the trauma, my life would remain on the same pattern it has prior to admission to the hospital” (#17) “having faith that new possibilities exist” (#6) “I realized that there could be other possibilities in life I could enjoy” (#3)
**A.4** Identity and Strengths	“After my recovery, I feel full from inside now, before it was emptiness” (#4) “I regained my ability to dream and this is a big achievement” (#7) “I’d say that my personality was markedly changed during this recovery process, I now see the difference between myself and the person who was admitted to the hospital 9 months ago, I used to be a very shy person who keeps to himself and absorbs abuses from others and accumulate bad thoughts and feelings to the limits of mental breakdown I’m no longer that” (#11) “ Being resilient has helped me a lot, it is important for people who have mental health issues to move on and keep going with all the struggles we face” (#8)
**A.5** Appreciation of life	“I feel better as I try again, I try to be better, I try to have dreams, I resist surrendering to death. I hated being dead where I’m still living” (#10) “. I started doing things I like again although in the beginning I wasn’t feeling happy to do it but with time, I regained my passion toward it” (#15)” I decided to do what I’m supposed to do as God wants me to be alive. when I feel down, I remember how I buried myself. I started to resist with my whole power and I feel scared to evoke my old identity” (#7) “Recovery means to feel the blessings you have even in the presence of struggles. It means you don’t feel disconnected” (#13) “Recovery means being able to live a better life, not just surviving, living. I know I’m recovering as I started to love myself” (#17) “I resist surrendering to death. I hated being dead where I’m still living. I started doing things I like again although in the beginning I wasn’t feeling happy to do it but with time, I regained my passion toward it. I decided to do what I’m supposed to do as God wants me to be alive” (#11) “When I feel down, I remember how I buried myself before, I resist with my whole power and I feel scared to evoke my old dead identity” (#7)
**B. Acceptance**	**“**The idea that relapse is possible, I’m not afraid of it” (#1) “Relapse can happen again, I think I’m prepared, I know what I should do” (#2) “I accepted my downs and relapses alongside doing my best” (#3) I accept all what I have been through either good or bad” (#1) “What I feel from my journey is I’m worthy of respect “(# 4) “In my views, I think recovery is acceptance, acceptance is the core of recovery and being free. The therapist has a role in teaching the patient how to accept his vulnerable human nature” (#15) “Recovery means accepting pain not avoiding it because avoiding it is inhuman and illogical” (#15) “Recovery means being able to deal with the pain, being able to receive and give love” (#5) “accepting one’s journey as it is and being able to deal with its reality” (#6) “I accepted my feelings (#3) “I can now forgive and let go of the past” (#7)
**C. Functional recovery**	“Recovery is to be able to complete your daily tasks and be up to your role in your family and surroundings, and able to engage with people around you” (# 2) “Work is important to me it is a big step” (# 9) "doing something useful in life is very good. By logic, what is its benefit (laughter), just to be happy in life without doing anything useful??? (#1) “it’s all about the ability to work, work is important” (# 10) “I think to recover from a mental disorder is achieved when a person can do his daily tasks and ease through the day without any disruption that comes from the mental disorder he suffers from. For me, waking up every morning, going to my work” (# 11) “I know that I’m improving when my interaction with people around me gets better, being able to create relationships and gain social skills and engage in many activities” (#12) “Recovery means that you can go on with life, do your duties, resume your activity” (#13) “I now go to work, I pray, I go to a dietitian, I go out weekly with my sister, and I go to gym. I have a strong will, there are lots of achievements I guess” (# 14) “I hated the disability resulting from mental illness, I know myself, I’m not that disabled, now I’m functioning, have a prestigious career, aspiring to do post-grad studies” (# 8)
**D. Hope**	“I love life despite its pain, I was sure my feelings, and my love of life can help me get through anything” (#7) “I’m still lonely and I still lack the full ability to communicate with people but I still have hope and dreams” (#7) “The recovery process gave me hope and an optimistic view on the future” (# 2) “Hope is the key; I never give up” (#8) “It is my ability to find hope” (# 6)
**E. Clinical recovery**	“I know I’m in recovery when I’m not doing any bizarre behavior or being aggressive” (# 12) “when I no longer experience symptoms” (# 9) “I feel like recovering when the somatic symptoms and nervousness decrease” (# 15) “I feel like I have improved 90% (that’s my parameter), when I feel good, no nervousness, no anxiety, no insomnia” (# 16) “Recovery means absence of most symptoms till 90%, I sometimes experience symptoms but not disabling like before” (# 8)

#### Theme 1: Domains of Posttraumatic Growth (PTG)

Inductive thematic analysis was used yet, 14 participants reported the five domains of PTG as identified by Tedeschi and Calhoun (2004). Participants did not report the domains as such, but rather what they shared could be classified into the five domains; relating to others, spirituality, new possibilities, appreciation of life, and personal identity and strengths [29].

##### Theme 1.1: Relating to others

Five participants described recovery as relating to others; the pain in the experience of mental health issues allowed participants to be compassionate with others.

“I now have compassion for others, I can connect with people, I was arrogant before the experience of mental illness. I now help people and engage in volunteer activities, the pain I went through gave me empathy for others” (#8)

##### Theme 1.2: Spirituality

Six participants described that their religious practices and the relationship with God helped their recovery journey, either by helping them to cope with mental health issues or through providing individuals with a sense of peace:

“Being a believer has a huge impact on me, it calmed me down after the constant anxiety and fear which were massive after discharge from the hospital” (#1)

“I was scared to deal with people, I had no idea what to do in life and work. After my spiritual relation with God, these fears haven’t disappeared completely but were less” (#13)

“I turned out to believe that I shouldn’t worry too much about everything, all what I should do is to work hard, God is there for me” (#6)

“The spiritual and religious practice have helped me in my journey so much” (#4)

“I’m close to god, I do the prayers that have helped me in my journey with the mental health problem” (#8)

##### Theme 1.3: New possibilities

Various positive changes associated with the experience of mental health issues were described by participants, such as experiencing recovery through finding new opportunities and options in life. For some, trauma experiences offered a different perspective. This theme was identified in the accounts of seven participants.

“My experience with the mental health issue made me see my options in life from a different perspective; I can now see the opportunities I have and I can use it all” (#8)

##### Theme 1.4: Appreciation of life

Six participants described recovery as appreciation and recognition of how precious life is:

“I resist surrendering to death. I hated being dead where I’m still living. I started doing things I like again although in the beginning I wasn’t feeling happy to do it but with time, I regained my passion toward it. I decided to do what I’m supposed to do as God wants me to be alive. When I feel down, I remember how I buried myself before, I resist with my whole power and I feel scared to evoke my old dead identity” (#7).

##### Theme 1.5: Personal identity and strength.

Four participants reported positive change in their identity during or after their recovery from mental health issues:

“I would say that my personality was markedly changed during this recovery process, I now see the difference between myself and the person who was admitted to the hospital 9 months ago, I used to be a very shy person who absorbs abuses from others and accumulate bad thoughts and feelings to the limits of mental breakdown I’m no longer that” (#11).

“After my recovery, I feel full from inside now, before it was emptiness” (#4).

#### Theme 2: acceptance

An overall of eight participants described recovery as acceptance; accepting human vulnerability and pain during the experience of mental health issue, and accepting the reality of the journey and being able to deal with it, and with one’s feelings:

“The idea that relapse is possible, I’m now not afraid of it” (#1)

“Relapse can happen again, I think I’m prepared, I know what I should do” (#2)

“I accepted my downs and relapses while doing my best” (#3)

#### Theme 3: functional recovery

Recovery as the ability to maintain both vocational and social functioning was described by nine participants:

“I think to recover from a mental disorder will be achieved when a person can do his daily tasks and ease through the day without any disruption that comes from the mental disorder he suffers from. For me, waking up every morning, going to my work” (#11).

#### Theme 4: finding hope

Four participants revealed a relationship between feeling of hope and recovery. Having hope in the future, and the ability to dream were associated with recovery.

“I love life now despite its pain, I was sure my feelings, and my love of life would help me get through anything. I’m still lonely and I still lack the full ability to communicate with people but I have hope and dreams” (#7)

“The recovery journey gave me hope and an optimistic view to the future” (#2)

#### Theme 5: clinical recovery

Five participants defined recovery in relation to symptom control:

“I know I’m in recovery when I’m not doing any bizarre behavior or being aggressive” (#12).

“Recovery means absence of most of the symptoms, I sometimes experience symptoms but not disabling like before” (#8).

## Discussion

The current paper aimed at exploring mental health recovery meaning informed by people who self-identify as having recovery experiences and living in the Egyptian community. Both personal and functional definitions of mental health recovery were stronger themes than clinical recovery in the accounts of the service users.

Domains of PTG were reported as both definitions and indicators of personal recovery in the current study. According to Tedeschi and Calhoun (2004), PTG is defined as positive psychological changes that occur after the experience of highly challenging life situation. Cognitive reconstruction to formulate a new identity is assumed to be underlying the process of PTG [30]. People with mental health concerns, including experiences of psychosis, are known to experience PTG [12, 31].

Findings from the current study support previous studies examining the experience of PTG in people with mental health issues [11, 21, 32]. Participants in the current study reported positive changes in the following domains; First, the subjective sense of belonging resulting from engagement in meaningful social relationships; actively choosing relationships, the ability to set boundaries for abusive and dysfunctional relationships, choosing to be assertive, and having the ability to empathize with others. All of these as participants reported are contributing factors to their engagement in meaningful relations.

Second, spirituality and religious practices were reported by participants. Families and people living with mental health issues in Egypt believe that having a mental health issues is a way of healing the soul. They return to God for prayers and asking for strengths and healing [33]. People with mental health issues may devote to religious commitments to make sense of their experience, or to rely on religious resources for coping [34]. Egyptian population are predominantly Muslim. There is a strong belief in God’s will which denotes high level of acceptance and optimism in healing [35]. Additionally, Muslims believe that suffering and illness are part of life and are not meaningless [36]. According to a scoping review, Gamieldien and colleagues found that spirituality is both a facilitator and indicator of mental health recovery in people with severe mental illness in LMICs [37].

Third, finding new possibilities and opportunities in life was revealed by participants in the current study; this may be related to the positive identity changes and recognizing that adversities could be positive transformations through reframing experiences with constructive and positive meanings. This corresponds with previous research findings [38–41]. Fourth, appreciation of life was attributed to the optimistic and positive appraisal of life, which developed over time and may be linked to the positive identity change. Fifth, narratives of recovery as reconstruction of identity and strengths in the current study correlate with the literature defining recovery process around the reawakening, rebuilding and utilization of a more positive sense of self [42].

Family members often support the cost of treatment when the functioning of individuals is impacted by mental health issues [43]. Due to low governmental expenditure on public health services [44], many Egyptians with mental health issues use private health services which is relatively costly. The late charismatic and popular Egyptian president Nasser once said “He who cannot support himself, cannot take his own decision” in reference to the value of employment, productivity, and financial independence [45]. This statement is popular in Egypt and highlights the value placed on productivity. This may account for the focus on functional recovery in accounts of mental health recovery as it may mitigate the costs families carry due to mental health issues.

Finding hope as reported by service users in this study has been found as an essential factor in promoting recovery from mental health issues in many studies [46, 47]. The final theme reported by participants in the current study was clinical recovery; symptoms of mental health issues are disabling and there is high stigma in Egypt [48, 49]. Therefore, supporting symptom remission should be supported if an individual express this as a treatment goal. Recovery meaning as conceptualized by participants in the current study is comparable to the conceptual framework of personal recovery which describes five recovery processes; Connectedness; Hope and optimism about the future; Identity; Meaning in life; and Empowerment (CHIME framework) which dominate in Anglophone countries [50]. All aspects of CHIME were supported in the current study except the empowerment domain. Egypt is among the collectivist cultures where group-favoritism dominate the out-group members culture [51]. Families and caregivers, in collaboration with mental health providers, often make treatment decisions without consultation with the person with mental health issues [52]. This is unlike mental health recovery accounts published in western cultures where individuality is emphasized [53]. People with mental health issues in Egypt continue to experience significant stigma and may be victimized through aggressive acts. therefore, There is a need to focus on the empowerment of people with mental health issues in Egypt to promote recovery and PTG [52].

## Strengths and limitations

The paper has a number of strengths; this is the first paper to explore definitions of recovery in community dwelling adults with mental health issues in Egypt. This will contribute to future development of interventions to support recovery and PTG in Egypt. Second, the recruitment of people with different mental health issues provides broader insight into the recovery experiences. This is important in the development of wider policy for the development of recovery-oriented mental health services in Egypt. Third, the identification of culturally-specific contributors of recovery contributes to further understanding of the contributions of culture in mental health recovery processes. Fourth, the use of field notes allowed the researcher to reflect upon participant responses and the triangulation of interview transcripts. Whilst the study had a small sample size, it allowed for the in-depth analysis of participant findings.

## Conclusion and implications

Despite the challenges in the delivery of mental health care in Egypt, mental health recovery meaning elicited from participants’ accounts in the current study can inform the design, implementation and wider policy change for recovery-oriented mental health practice in Egypt. Future research is required to delineate the role of PTG in mental health recovery in Egypt including; identification of whether PTG is perceived as a result of mental health issues, previous life trauma or both; identification of the proportion of individuals who have experienced PTG; factors that mediate the relationship between the experience of mental health issues and PTG; the role of families, friends and mental health professionals in promoting and supporting PTG and the training needs of these groups, and the development of manualized psychosocial interventions supporting PTG.

Finally, it is crucial to support local and national development of mental health service user groups to advocate effectively and have a proactive voice in mental health policy planning in Egypt [54].

## Data Availability

If someone wants to request the transcribed, anonymised data of this study, please contact the first author.

## References

[1] Lucianne P, Patterson S, O'Donovan A, Bradley G (2017). Protocol: A grounded theory of ‘recovery’—perspectives of adolescent users of mental health services.. BMJ open.

[2] Weinmann S, Koesters M (2016). Mental health service provision in low and middle-income countries: recent developments.

[3] Ghanem M, Mohsen Gadallah F. A, Meky S, Mourad G, Kholy El (2009). "National survey of prevalence of mental disorders in Egypt: preliminary survey". EMHJ-Eastern Mediterranean Health J.

[4] Fakhr El-Islam M (2008). Arab culture and mental health care.

[5] Fawzy,  M.E (2015). Quality of life and human rights conditions in a public psychiatric hospital in Cairo.

[6] Saeed S.I.N (2018). Aims of Egypt: Assessment of Governmental Mental Health System Egypt (2016–2017).

[7] Alanko A (2017). Improving mental health care: Finnish mental health policy rationale in the era of dehospitalisation.

[8] Borg M, Karlsson B (2017). Person-centredness, recovery and user involvement in mental health services.

[9] Pande A, El Shalakani A, Hamed A (2017). How can we measure progress on social justice in health care. The case of Egypt. Health Syst Reform..

[10] Calhoun LG, Cann A, Tedeschi RG, McMillan J (2000). A correlational test of the relationship between posttraumatic growth, religion, and cognitive processing. Journal of Traumatic Stress: Official Publication of The International Society for Traumatic Stress Studies.

[11] Slade M, Rennick-Egglestone S, Blackie L, Llewellyn-Beardsley J, Franklin D, Hui A, Thornicroft G (2019). Post-traumatic growth in mental health recovery: qualitative study of narratives. BMJ open.

[12] Ng F, Ibrahim N, Franklin D, Jordan G, Lewandowski F, Fang F, Roe D (2021). Post-traumatic growth in psychosis: a systematic review and narrative synthesis. BMC Psychiat..

[13] Tedeschi R.G, Calhoun L.G (2004). A clinical approach to posttraumatic growth.

[14] Anderson Clarke LM (2016). and B. Warner, Exploring recovery perspectives in individuals diagnosed with mental illness.

[15] Ibrahim N, Ghallab E, Ng F, Eweida R, Slade M. Perspectives on mental health recovery from Egyptian mental health professionals: A qualitative study. J Psychiatr Ment Health Nurs. 2022. 10.1111/jpm.12754. 10.1111/jpm.12754 33740825

[16] Ertekin Pinar S (2020). and S. Sabanciogullari, The relationship between functional recovery and quality of life in patients affected by schizophrenia and treated at a community mental health center in Turkey.

[17] de Wet A, Swartz L, Chiliza B (2015). Hearing their voices: The lived experience of recovery from first-episode psychosis in schizophrenia in South Africa.

[18] Slade M, Amering M (2008). and L. Oades, Recovery: an international perspective.

[19] Anthony WA.J.P.r.j., (1993). Recovery from mental illness: the guiding vision of the mental health service system in the 1990s. Psychosocial Rehabilitation J..

[20] Hickey J, Pryjmachuk S, Waterman H (2017). Exploring personal recovery in mental illness through an Arabic sociocultural lens.

[21] Bird V, Leamy M, Tew J, Le Boutillier C, Williams J, Slade M (2014). Fit for purpose? Validation of a conceptual framework for personal recovery with current mental health consumers. Aust N Z J Psychiatry..

[22] Husserl PJ (1997). Phenomenology and nursing.

[23] Dreyer P.S, Pedersen B.D.J.N.i (2009). Distanciation in Ricoeur's theory of interpretation: Narrations in a study of life experiences of living with chronic illness and home mechanical ventilation. Nurs Inq.

[24] Lernevall LST, Moi AL, Cleary M, Kornhaber R, Dreyer P (2020). Support needs of parents of hospitalised children with a burn injury: An integrative review. Burns..

[25] Phillippi J, Lauderdale J (2018). A guide to field notes for qualitative research: Context and conversation.

[26] Saunders B, Sim J, Kingstone T, Baker S, Waterfield J, Bartlam B, Burroughs H, Jinks C (2018). Saturation in qualitative research: exploring its conceptualization and operationalization. Qual Quant.

[27] de Cassia Nunes Nascimento L, de Souza TV, dos Santos Oliveira IC, de Moraes JRMM, de Aguiar RCB, da Silva LF (2018). Theoretical saturation in qualitative research: an experience report in interview with schoolchildren. Rev Bras Enferm.

[28] Clarke V, Virginia B. Teaching thematic analysis: Overcoming challenges and developing strategies for effective learning. Psychologist. 2013;26(2).

[29] Tedeschi RG, Calhoun LG (2004). Posttraumatic growth: conceptual foundations and empirical evidence. Psychol Inq..

[30] Coroiu A, Körner A, Burke S, Meterissian S, Sabiston CM (2016). Stress and posttraumatic growth among survivors of breast cancer: A test of curvilinear effects. Int J Stress Manag.

[31] Roe D, Miriam C, Abraham R. Persons with psychosis as active agents interacting with their disorder. Psychiatr Rehabil J. 2004;28(2):122. 10.2975/28.2004.122.128 15605747

[32] Wang X, Lee MY, Yates N (2019). From past trauma to post-traumatic growth: The role of self in participants with serious mental illnesses.

[33] Endrawes G, O’Brien L, Wilkes L (2007). Mental illness and Egyptian families.

[34] Pargament KI. The psychology of religion and coping: Theory, research, practice. New York: Guilford press; 2001.

[35] Ciftci A, Nev J, Corrigan PW. Mental health stigma in the Muslim community. J Muslim Ment Health. 2013;7(1).

[36] Merhej R (2019). Stigma on mental illness in the Arab world: beyond the socio-cultural barriers.

[37] Gamieldien F, Galvaan R, Myers B, Sorsdahl K (2020). Exploration of recovery of people living with severe mental illness (SMI) in low-income and middle-income countries (LMIC): a scoping review protocol. BMJ Open..

[38] Braehler C, Schwannauer M (2012). Recovering an emerging self: Exploring reflective function in recovery from adolescent-onset psychosis.

[39] Cadario E, Stanton J, Nicholls P, Crengle S, Wouldes T, Gillard M, Merry SN (2012). A qualitative investigation of first-episode psychosis in adolescents. Clin Child Psychol Psychiatry..

[40] Connell M, Schweitzer R, King R (2015). Recovery from first-episode psychosis and recovering self: A qualitative study.

[41] Kilkku N, Munnukka T, Lehtinen K (2003). From information to knowledge: the meaning of information-giving to patients who had experienced first-episode psychosis.

[42] Piat M, Sabetti J, Couture A, Sylvestre J, Provencher H, Botschner J, Stayner D (2009). What does recovery mean for me? Perspectives of Canadian mental health consumers. Psychiatr Rehabil J.

[43] Beekun RI, Hamdy R, Westerman JW, HassabElnaby HR (2008). An exploration of ethical decision-making processes in the United States and Egypt. J Bus Ethics.

[44] Abdel Ghafar A (2018). A stable Egypt for a stable region: Socio-economic challenges and prospects.

[45] Friddell B (2012). Afraid of Commitment: Gamal Abdel Nasser's Ephemeral Political Ideology-a New Definition of Nasserism.

[46] Bonney S, Stickley T (2008). Recovery and mental health: a review of the British literature.

[47] Corrigan PW, Salzer M, Ralph RO, Sangster Y, Keck L (2004). Examining the factor structure of the recovery assessment scale. Schizophr Bull.

[48] Sidhom E, Abdelfattah A, Carter JM, El-Dosoky A, Fakhr El-Islam MF (2014). Patients’ perspectives on stigma of mental illness (an Egyptian study in a private hospital). Front Psychiatry..

[49] Endrawes G, O'Brien L, Wilkes L (2007). Egyptian families caring for a relative with mental illness: A hermeneutic study.

[50] Leamy M, Bird V, Le Boutillier C, Williams J, Slade M (2011). Conceptual framework for personal recovery in mental health: systematic review and narrative synthesis. Br J Psychiatry..

[51] Darwish A-F, Huber GL (2003). Individualism vs collectivism in different cultures: a cross-cultural study. Intercult Educ.

[52] Loza N, Randa E (2017). Service users and carers in low-and middle-income countries. BJPsych Int..

[53] Slade M, Adams N, O'Hagan M (2012). Recovery: past progress and future challenges. Int Rev Psychiatry..

[54] Bird P, Omar M, Doku V, Lund C, Nsereko JR, Mwanza J, MHaPP Research Programme Consortium (2011). Increasing the priority of mental health in Africa: findings from qualitative research in Ghana, South Africa. Uganda and Zambia. Health Policy Plan..

